# Supplementary material for: Efficient Generation of Knock-In Zebrafish Models for Inherited Disorders Using CRISPR-Cas9 Ribonucleoprotein Complexes
Source: Int J Mol Sci. 2021 Aug 30;22(17):9429. doi: 10.3390/ijms22179429 (PMC8431507; doi:10.3390/ijms22179429)
Supplement: Supplementary file 1 [file ijms-22-09429-s001.zip › Supplemental document S1.pdf]

Detailed protocol of the development of knock-in zebrafish models.

A “shopping list” of reagents used is provided at the end of this document.

Step 1: gRNA design and efficacy test.

1.1 *sgRNA design*

Design of the sgRNAs is easily done using CRISPRscan (https://www.crisprscan.org) or CHOPCHOP (https://chopchop.cbu.uib.no) webtools with settings for Cas9 (PAM: NGG). When selecting the in-vitro T7 promotor, the tools immediate return sequences that include both the target specific region and the constant region for cloning-free gRNA synthesis and in-vitro transcription using T7 promotor. We prefer to add a 5’ clamp sequence (CCGCTAGG) to the target-specific oligonucleotide as the 5’end of the T7 promotor is very A-T rich.

Try to select a sgRNA with high predicted efficiency that directs Cas9 to cleave as close to the intended nucleotide substitution as possible. Order the target-specific oligo including constant region, and the sgRNA constant oligonucleotide (table S1) from your preferred oligonucleotide supplier (we use Sigma-Aldrich and IDT).

Note: If CRISPRscan or CHOPCHOP predict potential off-target binding-sites for a sgRNA, it can still be used as long as the off-target sites are not on the same chromosome. Potential off-target lesions are easily identified in the F1 generation by targeted sequencing, and can be removed by crossing with wildtype animals.

1.2 *Cloning-free sgRNA synthesis**

This method was first described by Gagnon et al (PMID: 24873830), and they provide a detailed description of the procedures in their supplementary datafiles. Note that we made some small adaptations. Figure A provides a graphical overview of the different sgRNA synthesis steps.

*In fact, we recently started to order commercially synthesized sgRNAs from IDT (link). Although not yet tested in knock-in studies, we found them to be cost-effective compared to the cloning-free sgRNA synthesis procedure. An order of 2 nmol is sufficient for dozens of injections, whereas the yield of cloning-free sgRNA synthesis is variable in our hands.

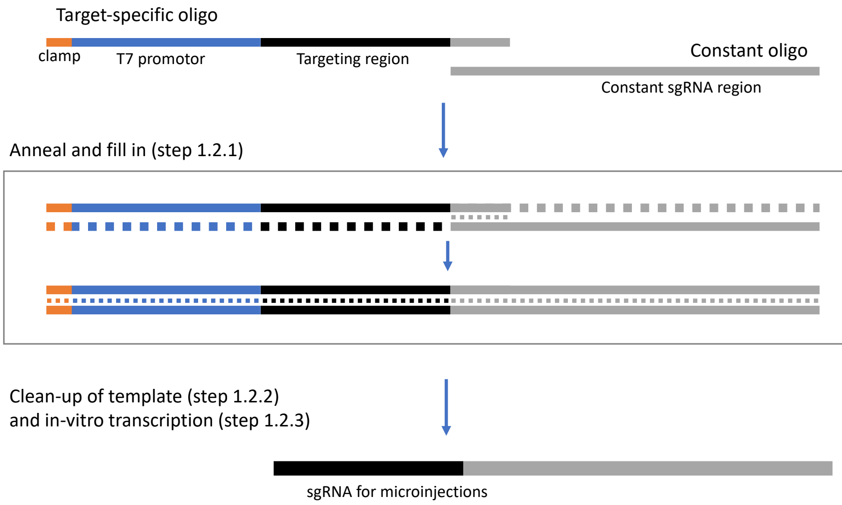

Figure A. schematic overview of sgRNA synthesis

Target-specific oligo: CCGCTAGCTAATACGACTCACTATA **-targeting sequence-** GTTTTAGAGCTAGAAATAGCAAG

Constant oligo: AAAAGCACCGACTCGGTGCCACTTTTTCAAGTTGATAACGGACTAGCCTTATTTTAACTTGCTATTTCTAGCTCTAAAAC

1.2.1 Oligonucleotide annealing and complementation (fill-in)

Oligonucleotides are dissolved at 100uM stock concentration, and kept at -20°C until use. We use Phusion polymerase (#M0530S, New England BioLabs) to anneal and complement the oligonucleotides. The following reaction mixture is prepared in a PCR tube for each sgRNA template:

**Component:**	
5x Phusion HF reaction Buffer	20ul
10mm dNTPs	2ul
target-specific oligonucleotide (100uM)	5ul
constant oligonucleotide (100uM)	5ul
Phusion(R) High-Fidelity Polymerase	2ul
MilliQ	66ul
Final volume	100ul

The reaction was sequentially incubated for 2 minutes at 98°C, 10 minutes at 50°C and 10 minutes at 72°C in a thermocycler. The reaction ensures the annealing of the oligonucleotides, and complements single-stranded overhangs. Alternative polymerases can be used, but make sure to choose one with similar proof-reading and end-tailing characteristics.

1.2.2 Oligonucleotide template purification

Annealed sgRNA templates were purified using the GenElute PCR clean-up kit (#NA1020, Sigma-Aldrich) precisely according to manufacturer's instructions with the exception that MQ water is used for the final elution step. To obtain a high concentration, we elute in 30ul instead of the indicated 50ul. Verify concentration by Nanodrop or similar.

Although most PCR clean-up kits can be used, we observed templates eluted with this kit perform better in the subsequent in vitro transcription reaction compared template purified with kits from some of the less-expensive manufacturers.

1.2.3 In-vitro transcription

The following reaction is prepared in DNase/RNase free PCR strips, using the MegaShortscript T7 kit (#AM1354, Invitrogen), and incubate o/n at 37°C in a thermo cycler. After the incubation, immediately proceed to the sgRNA purification describe in step 1.1.4.

Reagents	
ATP	2ul
GTP	2ul
CTP	2ul
UTP	2ul
T7 polymerase	2ul
MilliQ	6ul
sgRNA template (200-400ng)*	2ul
Buffer	2ul
Final volume	20ul

* In some occasions more input can increase sgRNA yield. In view of our relatively stable recovery of ~100-200ng/ul of sgRNA template from step 1.1.2, we usually use 2ul of sgRNA template as input.

1.2.4 sgRNA purification

SgRNAs were purified using the MEGAclear™ Transcription Clean-Up Kit (#AM1908, Invitrogen), aliquoted and stored at -80°C until use.

a) Bring the RNA sample to 100 µL with Elution Solution. Mix gently but thoroughly.
b) Add 350 µL of Binding Solution Concentrate to the sample. Mix gently by pipetting.
c) Add 250 µL of 100% ethanol to the sample. Mix gently by pipetting.
d) Apply the sample to the filter:
    - Insert a Filter Cartridge into 1 of the Collection and Elution Tubes supplied.
    - Pipet the RNA mixture onto the Filter Cartridge.
    - Centrifuge for 1 min at RCF 10,000–15,000 × g (typically 10,000–14,000 rpm). Spinning harder than this may damage the filters.
    - Discard the flow-through and reuse the Collection and Elution Tube for the washing steps.
e) Wash with 2 × 500µL Wash Solution.

Note: Make sure that the ethanol has been added to the Wash Solution Concentrate before using it.

- Apply 500µL Wash Solution. Draw the Wash Solution through the filter as in the previous step.
- Repeat with a second 500µL aliquot of Wash Solution.
- After discarding the Wash Solution, continue centrifugation for 1 min to remove the last traces of Wash Solution.

f) Elute RNA from the filter with 40µL Elution Solution using one of the methods described below; they are equivalent in terms of RNA recovery.
g) RNA elution
    - Place the Filter Cartridge into a new Collection/Elution Tube.
    - Apply 40µL of DNase/RNase free water to the center of the Filter Cartridge. Close the cap of the tube and incubate in a heat block set to 70°C 5–10 min.
    - Recover eluted RNA by centrifuging for 1 min at RT (RCF 10,000–15,000 × g).
    - To maximize RNA recovery, repeat this elution procedure with a second 40µL aliquot of DNase/RNase free water. Collect the eluate into a fresh tube. In the situation of high yields, the second elution is often in the range of concentrations that can be immediately used to prepare the sgRNA-Cas9 mixture
h) Assay RNA yield by nanodrop or similar.
i) Run 1 or 2ul recovered product on agarose gel to verify that a sgRNA of the appropriate length is formed. We normally use a normal DNA-type gel with EtBr dye, and a 100bp ladder. The sgRNA run usually just above the 100bp band of the ladder. Additional purification can be performed as described in the manual, but are typically not needed.
j) Aliquot in 5-10ul aliquots and store at -80°C until use.

1.3 sgRNA efficacy test

We typically first perform an injection with the sgRNA-Cas9 ribonucleoprotein(RNP) complexes without the template to assess their ability to induce on-target DNA cleavage. Although one could immediately add the template, we prefer to first determine the optimal sgRNA concentration for sufficient genome editing without toxic side effects of RNP injection. **We consider an sgRNA-Cas9 RNP as efficient when we observe evidence of genome editing by HRM in >70% of injected embryos.**

1.3.1 Microinjections

On the day before injections, prepare zebrafish for natural spawning according to facility procedures (separate males and females). Just prior to the injection, place males and females together in a spawning cage, and prepare the injection mixture as follows:

Reagents		
Alt-R® S.p. Cas9 Nuclease V3 (10ug/ul; # 1081058, IDT)*	0,32	ul
sgRNA**	100	ng/ul
KCl (2M stock)	0,55	ul
Phenol red (P0290, Sigma Aldrich)	0,8	ul
MQ water	…	ul
Final volume	4	ul

* Note that IDT also offers high fidelity Cas9, but this nuclease was not (yet?) function in zebrafish in our hands.
** 100ng/ul is our preferred starting concentration, but this can be decreased when sgRNA yield is low, or RNPs appear toxic.

Incubate the reaction mixture for 5 minutes at 37°C, fill the injection needle, and inject 1nl of mixture into the first cell of a fertilized zebrafish embryo. Preparation of the injection mixture can be done before placing the fish together. For most efficient genome-editing, we collect embryos for injection as early as possible (˜10 minutes after placing the fish together, a few pairs usually have produced sufficient eggs).

For sgRNA-Cas9 RNP efficacy tests, we typically inject 25-35 embryos per sgRNA, and raise them according to standard zebrafish procedures. Make sure to keep several uninjected controls to be able to relate potential poor survival to batch quality, in the injection procedure, and as wildtypes in the genetic analysis.

1.3.2 Analysis of RNP activity

One day post-fertilization and injection, embryos are collected individually in PCR strips. After removing all liquid, 25ul of lysis buffer (25mM NaOH, 0.2mM EDTA) is added to each embryo. Samples are incubated in a thermocycler at 95oC for 20 minutes, after which the samples are neutralized with 2.5ul of 1M Tris pH7.5 (mix several times by pipetting to dissociate any remaining lumps of tissue), and dilute 5-10x with MQ water to obtain input for PCR and HRM analysis.
Note that PCR protocols are target-specific, and at the user's discretion. Any high-fidelity polymerase should work. We prefer Phusion or Q5 polymerase (#M0530S, New England Biolabs), and sometimes adjust sample dilutions to improve amplification efficiency. For the optimal HRM results, amplicons <150bp (or even <100bp) are recommended. See also Thomas et al (PMID: 25503746) for more details on the HRM method and primer/amplicon design.

For the HRM analysis, prepare the following PCR reaction for all samples in the appropriate qPCR vessels. We typically analyze 8-12 injected embryos per RNP, and 4 uninjected wildtypes.

Reagent	
5x Phusion HF reaction Buffer	4ul
dNTPs (10mM solution)	0,4ul
forward primer (10uM solution)	0,5ul
reverse primer (10uM solution)	0,5ul
EvaGreen Dye (#31000, Biotium)	1ul
Phusion polymerase	0,2ul
sample (5x diluted)	1ul
MQ water	xx
Final volume	20ul

We normally perform both the PCR step and HRM step as a single protocol in a Quantstudio 3 Real-Time PCR System (Applied Biosystems). Our thermocycler protocol is provided below in Figure B.

Be aware of the melting and rapid cooling (maximum ramp rate) after the final extension step (arrows), and the low ramp rate (0,1°C/sec) during the melting step. Annealing temperature (PCR stage step 2) is at the user's discretion.

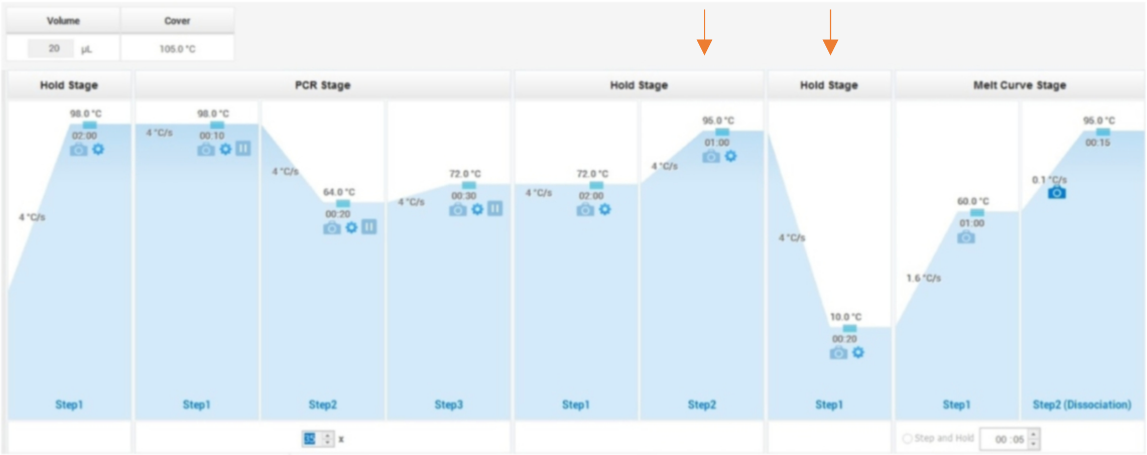

Figure B: HRM thermocycler protocol on a Quantstudio 3 Real-Time PCR System

We also performed successful HRM analyses using the BioRad's CFX96, and QiaGen Rotor-Gene Q. If needed, the amplification steps can be done in a regular PCR machine, and later transfer sample to the appropriate vessel and machine for the HRM analysis. Do not forget the final melting and cooling steps, they are not part of a regular PCR protocol, but crucial for the HRM. HRM analysis was not successful when using standard qPCR dyes such as SybrGreen or GoTaq.

A typical HRM result obtained from wildtype embryos (on unsuccessfully injected embryos) looks like a single melting peak. All amplicons are similar, and show the same melting-behaviour. Genome-editing signatures can look like double peak, multiple (often smaller) peaks or shoulder peaks. This is not one typical signature of success. Sanger sequencing should be used to confirm that non-wildtype HRM peaks are indicative of genome editing events. In a sequencing read obtained from an "edited" embryo, double peaks can be observed close to the sgRNA target site. Cleanup of the sample with a PCR cleanup kit is recommended prior to sequencing.
Typical outcomes of HRM and Sanger sequencing are provided in figure 1C and 1D of the manuscript.

Step 2: RNP and template injections

After the identification of an efficient sgRNA-Cas9 RNP complex (see step 1.3), we proceed with the injections with both RNP and antisense asymmetric DNA oligo template. Another advantage of first identifying the optimal sgRNA is that now a template can be ordered with the appropriate silent PAM-site change (if possible) to avoid re-cleaving of a knock-in allele. We observed somatic knock-in events in 3.4% - 18.0% of sequencing reads, of which the lowest value already correlated with germline transmission of the variant of interest in 30% of injected adults. **We therefore recommend to raise embryos from an injection with a somatic knock-in events in >3% of sequencing reads (obtained from a pool of embryos).**

2.1 Antisense asymmetric DNA oligo template design

The asymmetric antisense oligonucleotide templates are comprised of a 90-nucleotide arm and a 36-nucleotide arm calculated from the Cas9 cleavage position. The short arm is complementary to the sequence upstream of the Cas9 cleavage position, the long arm complementary to the downstream region. Note that the sgRNA-Cas9 RNP complex can target either DNA strand, and cleavage position is always determined as 3-4 nucleotides upstream of the PAM site (NGG for S.p.Cas9). Variants of interest were introduced in the short arm, PAM site variants in the long arm*. The 12-nt deletion ripor2 template was designed by removing these specific 12 nucleotide from the template, effectively reducing the length of the short arm. Oligonucleotide templates can be ordered from your preferred oligonucleotide supplier (we use Sigma-Aldrich and IDT).

* This was how we designed the templates based on the options for sgRNA design. Crossover during homology directed repair was likely the reason we obtained also reads with the variant of interest, but without PAM site change (and vice versa). As such, variants can (in all likelihood) also be on the same arm, or switched, as long as the variants are close to the cleavage site for maximum knock-in efficiency. There is ample literature on CRISPR technology to consult for more information.

2.2 Microinjections with RNP and template (and xrcc6-targeting PMO)

Here we use the following injection mixture that furthermore includes the asymmetric antisense oligonucleotide template and the *xrcc6*-targeting PMO (optional).

Prepare zebrafish for natural spawning according to standard procedures. Just prior to the injection, prepare the injection mixture as follows:

Reagents		
Alt-R® S.p. Cas9 Nuclease V3 (10ug/ul; # 1081058, IDT)	0,4	ul
sgRNA	100	ng/ul
Asymmetric antisense oligonucleotide template (dissolved in MQ water)	1uM	(final concentration in injection mixture)
xrcc6-targeting PMO (25ug/ul)	0,3	ul (optional)
KCl (2M stock)	0,69	ul
Phenol red (P0290, Sigma Aldrich)	1	ul
MQ water	…	ul
Final volume	5	ul

Incubate the reaction mixture for 5 minutes at 37°C, fill the injection needle, and inject 1nl of mixture into the first cell of a fertilized zebrafish embryo.

2.3 Analysis of knock-in efficiency with massive parallel sequencing.

When knock-in-specific restriction analyses or PCR strategies are available, they can be used to obtain a first indication of knock-in efficiency. However, these approaches have not always been very reliable and reproducible in our hands. Therefore, we immediately proceed with massive parallel sequencing of the target region in a pool of injected embryos. Advantage of this approach is that we obtain insight in the proportion of correct genome-editing within the sample. We used the IonTorrent platform, but Illumina or PacBio platforms are equally suitable.

For this approach we pooled 20-25 injected embryos at 1 day post fertilization and injection. DNA was extracted using QIAamp DNA Mini Kit (#51304, QIAGEN) according to the manufacturer's instruction. We elute the DNA in 75ul elution buffer. Key in this step is to obtain high-purity genomic DNA, the QIAamp kit is just one of the many options.

We recommend to design an PCR strategy that is different from the HRM, with an amplicon length of 200-300bp. We typically obtain a yield of 50-100ng/ul of genomic DNA, and use 2ul as input in a 40ul PCR reaction. A higher reaction volume compared to common practice is recommended to immediately obtain sufficient input for sequencing (in our case: ~250ng/sequencing sample). After confirmation of the PCR product using gel electrophoresis, prepare samples for sequencing according to the guidelines of the local sequencing service facility or provider.

For each platform that leads to sufficient sequencing reads, use of barcoded primers (see table S1) and pooling of the amplicons, is a good strategy to reduce costs. Pooling up to 6 samples on the IonTorrent Platform always returned >50,000 reads per target.

2.4. *Analysis of knock-in efficiency*

The analysis of knock-in efficiency largely depends on the platform and procedures of the local sequencing facility. In our studies, the obtained sequencing reads were demultiplexed, mapped against wildtype specific Fasta files, and analyzed for knock-in events using the SEQNEXT software (JSI Medical Systems, Ettenheim, Germany).

Specifically for SEQNEXT, the software allows the visualization of individual reads per sample and target gene (right-click on the representative sequence and select "show reads"). This opens in window in which you can scroll through all sequencing reads. By right-clicking on individual nucleotides in any of the reads, it is possible to add them to a filter allowing the visualization of all reads with or without this nucleotide. In the left lower corner, the software provides a percentage of reads that meet the selected filtering criteria. Using this approach, we filtered for either wildtype reads, correct knock-in reads with PAM site change, etc. For convenience's sake, we filtered 20bp up- and downstream of the variants of interest. Variants beyond these regions were generally single nucleotide changes, insertions or deletions with a very low frequency, and therefore deemed sequencing errors.

With this approach, we observed somatic knock-in events in 3.4% - 18.0% of sequencing reads. Larvae raised from the batch in which we observed 3.4% of reads with correct knock-in events already led to the identification of 3 founder fish (2 without *in cis* PAM-site change) out of the 10 injected adults that were screened. **As such, we recommend to raise only batches of injected embryos in which somatic knock-in events are identified in >3% of sequencing reads to adulthood** to allow for the swift identification of founder fish (see step 3). In case of low somatic knock-inefficiency, the *xrcc6*-targeting PMO can be added to the sgRNA-Cas9 RNP and template injection mixture in step 2.2.

Step 3: identification of germ-line positive founders

After raising the injected embryos according to standard facility procedures, adults can be fertile as early as 2.5 months post fertilization. To identify animals with germline transmission, we breed injected adults with strain-matched wildtype zebrafish. After embryos are collected, make sure that the injected adults can be individually identified either by temporary housing in single boxes, Vie-tagging (PMID: 33652779) or other institutionally approved methods.

At 1 day post fertilization, pool 20-25 embryos per clutch, and extract DNA as described in step 2.3. *Optional*: raise the remaining embryos pending the sequencing results.
Perform the same PCR as used to determine the somatic knock-in efficiency, and submit the obtained amplicons to massive parallel sequencing. When barcodes are used, samples from embryos of 5-10 breeding pairs can be easily pooled to reduce costs. Identification of the variant of interest in the genomic DNA of the embryos will pinpoint the adults that carry the variant in their germline.
After raising the F1 generation (outcross) from a germline positive founder, we employ standard genotyping with Sanger sequencing to identify F1 zebrafish that are heterozygous for the variant of interest. We always cross these fish with a strain-matched wildtype once more before we breed homozygous animals for phenotypic analysis.

Table: "shopping list" of required reagents and preferred suppliers.

reagent	catalog number	supplier
sgRNA oligonucleotide templates*	custom	Sigma Aldrich or IDT
Constant oligo: AAAAGCACCGACTCGGTGCCACTTTTTCAAGTTGATAACGGACTAGCCTTATTTTAACTTGCTATTTCTAGCTCTAAAAC		
Target-specific oligo: CCGCTAGCTAATACGACTCACTATA **-targeting sequence-** GTTTTAGAGCTAGAAATAGCAAG		
Phusion high-fidelity polymerase*	M0530S	New England BioLabs
GenElute PCR clean-up kit*	NA1020	Sigma-Aldrich
MegaShortscript T7 kit*	AM1354	Invitrogen
MEGAclear™ Transcription Clean-Up Kit*	AM1908	Invitrogen
Alt-R® S.p. Cas9 Nuclease V3 (10ug/ul)	1081058	IDT
2M KCl solution	Dissolve cell culture grade KCl powder, filter sterilize	
Phenol red solution	P0290	Sigma-Aldrich
EvaGreen Dye	31000	Biotium
Asymmetric antisense oligonucleotide template	custom, regular DNA oligonucleotide	IDT
xrcc6-targeting PMO	custom PMO: ACTTTTTAGGCTCACCTGCATAGT	Gene-Tools

In addition, standard molecular biology laboratory equipment (thermocycler, qPCR machine, gel-electrophoresis equipment, thermomixer, centrifuge), and a zebrafish microinjection setup are needed to replicate the protocol.

*Alternatively, ready-to use Alt-R® CRISPR-Cas9 sgRNAs can be ordered from IDT.
