# Supplementary material for: Efficient Generation of Knock-In Zebrafish Models for Inherited Disorders Using CRISPR-Cas9 Ribonucleoprotein Complexes
Source: Int J Mol Sci. 2021 Aug 30;22(17):9429. doi: 10.3390/ijms22179429 (PMC8431507; doi:10.3390/ijms22179429)
Supplement: Supplementary file 1 [file ijms-22-09429-s001.zip › Supplemental figure S1.pdf]

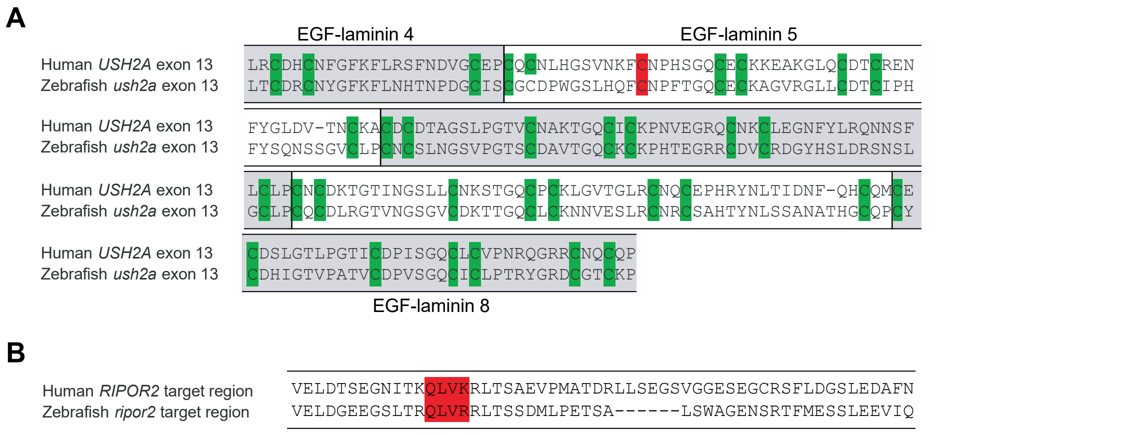

Supplemental Figure S1. A) Sequence alignment of the amino acids encoded by human and zebrafish *USH2A* exon 13 reveals a high conservation at both the amino acid level, and the EGF-laminin protein domain architecture. Conserved cysteines that form the disulphide bonds in the EGF-laminin domains are indicated in green. The frequently mutated cysteine at position p.759 of human usherin, and orthologous cysteine of zebrafish usherin, are indicated in red. **B**) Sequence alignment of the amino acids encoded by human and zebrafish *RIPOR2* target region in exon 14. The amino acids corresponding to human RIPOR2 p.(Gln566_Lys569del) are indicated in red.
