# Supplementary material for: Efficient Generation of Knock-In Zebrafish Models for Inherited Disorders Using CRISPR-Cas9 Ribonucleoprotein Complexes
Source: Int J Mol Sci. 2021 Aug 30;22(17):9429. doi: 10.3390/ijms22179429 (PMC8431507; doi:10.3390/ijms22179429)
Supplement: Supplementary file 1 [file ijms-22-09429-s001.zip › Supplemental table S1.pdf]

<u>Table S1:</u> Target-specific primers with or without barcodes for multiplex sequencing.

sgRNA constant oligo	AAAAGCACCGACTCGGTGCCACTTTTTCAAGTTGATAACGGACTAGCCTTATTTTAACTTGCTATTTCTAGCTCTAAAAC
sgRNA *ush2a* C771F targeting oligo	CCGCTAGCTAATACGACTCACTATA<u>GAGTGAAGGGATTACAGAAC</u>GTTTTAGAGCTAGAAATAGCAAG
ush2a C771F template	TTGTAACTATGGCTTCAAATTCCTCAATCACACCAATCCCGATGGTTGCATTTCCTGTGGCTGTGACCCATGGGGTTCTCTGCATCAGTTCTTTAATCCCTTCACTGGACAGTGTGAGTGCAAAGC
ush2a HRM_fw	TGGCTTCAAATTCCTCAATCA
ush2a HRM_rv	AGGCAGACTCCACTGCTGTT
sgRNA *ripor2* del12 targeting oligo	CCGCTAGCTAATACGACTCACTATA<u>GGACCAGGCAGCTGGTGAGG</u>GTTTTAGAGCTAGAA
ripor2 del12 template	CTCCAGACTGCTCTCCATAAAAGTCCTGCTGTTCTCTCCAGCCCAGCTCAGAGCACTGGTCTCAGGCAGCATATCAGACGAGGTCAGTCTCCTGGTCAGACTGCCTTCCTCCCCATCCAG
ripor2 HRM_fw	ACCAGAGGACGTGGTGTTTC
ripor2 HRM_rv	CACTGGTCTCAGGCAGCATA
sgRNA *tp53* R217H targeting oligo*	CCGCTAGGTAATACGACTCACTATAGG<u>GCAATAGCAGCTGCATGGGG</u>GTTTTAGAGCTAGAAATAGC
tp53 R217H template	AGTTCACAAGAGGAGGAATCAAATATGCAGTACTTACTCCTGAGTCTCCAGAGTGATGATTGTGAGGATGGGCCGGTGGTTCATGCCCCCCATGCAGCTGCTATTGCACATGTAGTTTAGTAGCAC
tp53 HRM_fw / IONT_F	AAATTGCCAGAGTATGTGTCTGTCC
tp53 HRM_rv / IONT_R	ATGAGAGCAGCATCATGAAGCAT
*ush2a*_IONT_F	AGGCAGACTCCACTGCTGTT
*ush2a*_IONT_R	TTTCTCCACCAACAGAATCT
*ush2a*_IONT_barcode1_F	<u>CGATGT</u>AGGCAGACTCCACTGCTGTT
*ush2a*_IONT_barcode1_R	<u>CGATGT</u>TTTCTCCACCAACAGAATCT
*ush2a*_IONT_barcode2_F	<u>CTTGTA</u>AGGCAGACTCCACTGCTGTT
*ush2a*_IONT_barcode2_R	<u>CTTGTA</u>TTTCTCCACCAACAGAATCT
*ush2a*_IONT_barcode3_F	<u>ATCACG</u>AGGCAGACTCCACTGCTGTT
*ush2a*_IONT_barcode3_R	<u>ATCACG</u>TTTCTCCACCAACAGAATCT
*ush2a*_IONT_barcode4_F	<u>ACTTGA</u>AGGCAGACTCCACTGCTGTT
*ush2a*_IONT_barcode4_R	<u>ACTTGA</u>TTTCTCCACCAACAGAATCT
*ush2a*_IONT_barcode5_F	<u>GATCAG</u>AGGCAGACTCCACTGCTGTT
*ush2a*_IONT_barcode5_R	<u>GATCAG</u>TTTCTCCACCAACAGAATCT
*ush2a*_IONT_barcode6_F	<u>GGCTAC</u>AGGCAGACTCCACTGCTGTT
*ush2a*_IONT_barcode6_R	<u>GGCTAC</u>TTTCTCCACCAACAGAATCT
*ush2a*_IONT_barcode7_F	<u>ATGTCA</u>AGGCAGACTCCACTGCTGTT
*ush2a*_IONT_barcode7_R	<u>ATGTCA</u>TTTCTCCACCAACAGAATCT
*ripor2*_IONT_F	ACCAGAGGACGTGGTGTTTC
*ripor2*_IONT_R	CACAGAGAGCTCATTCACCTTG

*ripor2*_IONT_barcode1_F	CGATGTACCAGAGGACGTGGTGTTTC
*ripor2*_IONT_barcode1_R	CGATGTCACAGAGAGCTCATTCACCTTG
*ripor2*_IONT_barcode2_F	CAGATCACCAGAGGACGTGGTGTTTC
*ripor2*_IONT_barcode2_R	CAGATCCACAGAGAGCTCATTCACCTTG
*ripor2*_IONT_barcode3_F	CTTGTAACCAGAGGACGTGGTGTTTC
*ripor2*_IONT_barcode3_R	CTTGTACACAGAGAGCTCATTCACCTTG
*ripor2*_IONT_barcode4_F	TTAGGCACCAGAGGACGTGGTGTTTC
*ripor2*_IONT_barcode4_R	TTAGGCCACAGAGAGCTCATTCACCTTG
*ripor2*_IONT_barcode5_F	GATCAGACCAGAGGACGTGGTGTTTC
*ripor2*_IONT_barcode5_R	GATCAGCACAGAGAGCTCATTCACCTTG
*ripor2*_IONT_barcode6_F	TAGCTTACCAGAGGACGTGGTGTTTC
*ripor2*_IONT_barcode6_R	TAGCTTCACAGAGAGCTCATTCACCTTG
*ripor2*_IONT_barcode7_F	GGCTACACCAGAGGACGTGGTGTTTC
*ripor2*_IONT_barcode7_R	GGCTACCACAGAGAGCTCATTCACCTTG
*tp53*_IONT_barcode1_F	CGATGTAAATTGCCAGAGTATGTGTCTGTCC
*tp53*_IONT_barcode1_R	CGATGTATGAGAGCAGCATCATGAAGCAT
*tp53*_IONT_barcode2_F	CTTGTAAAATTGCCAGAGTATGTGTCTGTCC
*tp53*_IONT_barcode2_R	CTTGTAATGAGAGCAGCATCATGAAGCAT
*tp53*_IONT_barcode3_F	ATCACGAAATTGCCAGAGTATGTGTCTGTCC
*tp53*_IONT_barcode3_R	ATCACGATGAGAGCAGCATCATGAAGCAT
xrcc6-targeting PMO	ACTTTTTAGGCTCACCTGCATAGT

Underlined sequences are either the gRNA sequence (in sgRNA oligos), or the barcode sequence (in Ion Torrent sequencing oligos).

*a 22nt sgRNA appears to be used by Prykhozhij *et al*., sequence was ordered and used as indicated in their publication [4]
